# Overlap of vitamin A and vitamin D target genes with CAKUT-related processes

**DOI:** 10.12688/f1000research.51018.2

**Published:** 2022-04-21

**Authors:** Ozan Ozisik, Friederike Ehrhart, Chris T. Evelo, Alberto Mantovani, Anaïs Baudot

**Affiliations:** 1 Aix Marseille University, Inserm, MMG, Marseille, 13385, France; 2 Department of Bioinformatics - BiGCaT, Maastricht University, Maastricht, 6200 MD, The Netherlands; 3 Department of Bioinformatics, NUTRIM/MHeNs, Maastricht University, Maastricht, 6200 MD, The Netherlands; 4 Istituto Superiore di Sanità, Rome, 00161, Italy; 5 Barcelona Supercomputing Center (BSC), Barcelona, 08034, Spain

**Keywords:** CAKUT, vitamin A, vitamin D, nutrients, environmental factor, renal development

## Abstract

Congenital Anomalies of the Kidney and Urinary Tract (CAKUT) are a group of abnormalities affecting the kidneys and their outflow tracts. CAKUT patients display a large clinical variability as well as a complex aetiology. Only 5% to 20% of the cases have a monogenic origin. It is thereby suspected that interactions of both genetic and environmental factors contribute to the disease. Vitamins are among the environmental factors that are considered for CAKUT aetiology. In this study, we aimed to investigate whether vitamin A or vitamin D could have a role in CAKUT aetiology. For this purpose we collected vitamin A and vitamin D target genes and computed their overlap with CAKUT-related gene sets. We observed limited overlap between vitamin D targets and CAKUT-related gene sets. We however observed that vitamin A target genes significantly overlap with multiple CAKUT-related gene sets, including CAKUT causal and differentially expressed genes, and genes involved in renal system development. Overall, these results indicate that an excess or deficiency of vitamin A might be relevant to a broad range of urogenital abnormalities.

## Introduction

Congenital Anomalies of the Kidney and Urinary Tract (CAKUT) are a group of abnormalities affecting the kidneys and their outflow tracts, including the ureters, the bladder, and the urethra. In the European Union, the overall prevalence of CAKUT (in live plus stillbirths) between 2011 and 2018 was approximately 35:10,000. ^1^ The main anomalies observed in CAKUT are hydronephrosis (13.02:10,000) and multicystic renal dysplasia (4.33:10,000); these are followed by posterior urethral valves (1.26:10,000), bilateral renal agenesis (1.27:10,000) and bladder exstrophy/epispadia (0.62:10,000); many other anomalies with low prevalence can also be observed. ^1^ This clinical variability observed in patients is accompanied by a variable severity of the phenotypes. ^2^

Approximately 40 different genes are known to be associated with monogenic causes of CAKUT in humans, but they explain only 5% to 20% of the cases. ^3, 4^ The clinical variability and the complex aetiology of CAKUT cases suggest a multifactorial origin with complex interactions of both genetic and environmental factors contributing to the disease. ^2, 3, 5^

The role of different environmental factors in CAKUT pathogenesis has been studied previously. It has been shown, for instance, that the drugs inhibiting the angiotensin-converting enzymes cause a specific form of CAKUT, namely tubular dysgenesis. ^6^ Many prenatal and maternal factors were also assessed for their involvement in CAKUT and related anomalies. For example, studies have observed CAKUT associations with pregestational maternal diabetes mellitus, ^7, 8, 9^ gestational maternal diabetes mellitus, ^8, 10, 11^ abnormal volume of amniotic fluid, ^7, 10^ urogenital infections before the pregnancy, ^12^ any infection during the pregnancy, ^12^ maternal overweight and obesity, ^7, 8, 11, 13^ and lower infant birth weight. ^7, 8, 9, 10^ These maternal and prenatal factors are modulated by environmental risk factors. In particular, maternal diabetes and overweight/obesity as well as low birth weight are related to the nutrition of the maternal-fetal dyad. In this regard, trace nutrients, like vitamins, are likely to play important roles that still await full characterization. ^14^

Vitamins are known to regulate renal development. ^15, 16, 17, 18^ The association of maternal vitamin A deficiency with nephron reduction was studied on a rat model. ^19^ The inactivation of the retinoic acid nuclear receptors also resulted in renal malformations in mice. ^20^ An additional study on rats showed that vitamin A deficiency downregulates RET expression which is essential for epithelial-mesenchymal interaction during renal development. ^21^ Goodyear *et al*. compared a group of pregnant women in Bangalore with a high (55%) prevalence of vitamin A deficiency with a group of pregnant women in Montreal with negligible vitamin A deficiency. ^22^ The authors found that renal volume in newborns, adjusted for body surface area, was significantly lower in the vitamin A-deficient population. While the two populations are different for other genetic and environmental reasons, these results suggest that vitamin A deficiency may have a role in human CAKUT. El-Khashab *et al*. reported a similar finding in their study on a cohort of Egyptian mother-child pairs; ^23^ children of vitamin A deficient mothers had significantly lower kidney sizes.

The research on the effects of vitamin D deficiency on kidney development has generated interesting results. Rogers *et al*. ^24^ observed that incubation of metanephroi with vitamin D3 prior to implantation into adult rats increased the number of glomeruli. Conversely, Maka *et al*. ^25^ and Nascimento *et al*. ^26^ observed that maternal vitamin D deficiency stimulated nephrogenesis in rats. Following these studies, Boyce *et al*. examined the long term effects of maternal vitamin D deficiency and observed that adult male rat offspring of vitamin D deficient dams had reduced creatinine clearance, indicating reduced renal functional capacity. ^27^ They also observed a significant upregulation of renal renin mRNA expression in fetuses and adult male offspring, and reported smaller kidneys in the offspring. Miliku *et al*. examined the association of 25-hydroxyvitamin D levels during mid-pregnancy with childhood kidney outcomes among 4212 mother-child pairs. ^28^ They observed that children of mothers who were vitamin D deficient during pregnancy had a larger combined kidney volume compared to children of mothers who had optimal vitamin D levels.

In this study, we examined whether vitamin A and vitamin D target genes are related to gene sets involved in CAKUT and renal system development. Our aim is to generate hypotheses for the involvement of these vitamins in the disease aetiology. We first created a list of vitamin A and vitamin D target genes, extracting information from the Comparative Toxicogenomics Database (CTD) ^29^ as well as from two publications. ^30, 31^ We then constructed different gene sets relevant to CAKUT, including genes mutated in monogenic forms of CAKUT, genes involved in renal system development, and genes involved in different pathways of interest. Finally, we performed overlap analyses to identify the genes involved in these different gene sets as well as the significant overlaps.

## Methods

### Vitamin A target genes

We first queried vitamin A and downloaded all gene interactions of vitamin A and its descendants from the Comparative Toxicogenomics Database (CTD). ^29^ We selected only the interactions supported by at least two references. This gave a list of 1086 target genes.

As an independent source, we used the data from the study of Balmer and Blomhoff. ^30^ In this study, the authors reviewed published data from 1191 articles to identify genes regulated by retinoic acid (the active metabolite of vitamin A) in humans and other species. Only a small subset of these articles are part of the CTD curation. The authors provide a list of retinoic acid target genes split into four categories, ranging from strong evidence to indirect regulation through a transcriptional intermediary. We selected the genes from all of these categories, converted gene symbols to human orthologs, and updated gene symbols when necessary using the HUGO Gene Nomenclature Committee (HGNC), Rat Genome Database (RGD), Mouse Genome Database (MGD), BioMart and SynGo. The final list of genes obtained from Balmer and Blomhoff contains 521 target genes, of which 229 are common with genes from CTD.

### Vitamin D target genes

We queried vitamin D and downloaded all gene interactions of vitamin D and its descendants from the CTD. We selected only the interactions supported by at least two references, and obtained a list of 263 target genes.

Ramagopalan *et al*. ^31^ identified 230 genes with significant expression changes in response to vitamin D on lymphoblastoid cell lines using microarrays. This publication is not part of the CTD curation, hence it brings additional and independent information. We checked the gene names using HGNC and obtained a list of 210 target genes.

There are only 15 common genes between the list from CTD and the list from Ramagopalan *et al*. The combined list of vitamin D target genes (458 genes) has 134 genes in common with the combined list of vitamin A target genes (1378 genes).

### CAKUT-related gene sets

We collected complementary gene sets relevant to CAKUT and renal system development from several sources, outlined below.

**CAKUT causal genes set**

First, we created a set of genes known to be mutated in the monogenic form of CAKUT. To do so, we combined two lists of genes provided in the studies of van der Ven *et al.* ^3, 4^ Only six genes are different between these two lists, and their union leads to 42 causal genes (see *Extended data* ^32^).

**CAKUT differentially expressed genes set**

Jovanovic *et al.* ^33^ identified 27 upregulated and 51 downregulated genes as a result of the transcriptome profiling of 15 CAKUT and 7 control ureter samples. We retrieved these differentially expressed genes (DEGs) from the supplementary file they provided. We updated gene names with the help of HUGO Gene Nomenclature Committee (HGNC) and obtained a final list of 74 DEGs (see *Extended data* ^32^).

**Gene Ontology kidney development gene sets**

We extracted the genes annotated with the three terms below from Gene Ontology (GO). ^34, 35^ Please note that these terms have parent-child relationships.

• GO:0072001 Renal system development (315 genes)
• GO:0001822 Kidney development (306 genes)
• GO:0060993 Kidney morphogenesis (96 genes)

**Reactome pathways**

We selected different pathways of interest for CAKUT. The renin-angiotensin system is essential for kidney development and mutations in the genes of this system result in CAKUT. ^36^ In addition, Davis *et al.* discussed the role of RET signaling in kidney development and CAKUT. ^37^ In multiple studies ^38– 43^ the roles of WNT, NOTCH, and Hedgehog signaling in kidney development and kidney diseases are discussed. Based on these studies we selected the following Reactome ^44^ pathways:

• R-HSA-2022377 Metabolism of angiotensinogen to angiotensins (18 genes)
• R-HSA-8853659 RET signaling (38 genes)
• R-HSA-195721 Signaling by WNT (328 genes)
• R-HSA-157118 Signaling by NOTCH (233 genes)
• R-HSA-5358351 Signaling by Hedgehog (149 genes)

**WikiPathways**

From WikiPathways Rare Disease Portal ^45^ we obtained the following four CAKUT-related pathways:

• WP5053 Development of ureteric collection system (47 genes)
• WP4823 Genes controlling nephrogenesis (43 genes)
• WP5052 Nephrogenesis (17 genes)
• WP4830 GDNF/RET signaling axis (23 genes)

### Overlap analyses

We computed the overlaps between vitamin target gene sets and the CAKUT gene sets of interest defined previously. After obtaining the overlap results, we calculated the significance of the overlaps using the hypergeometric test. Hypergeometric test calculates the statistical significance of getting k successes in n draws without replacement from a finite population of size N that contains a total of K successes. In our context, k is the number of genes overlapping with a specific gene set, K is the size of that gene set, n is the number of vitamin targets, and N is the background gene set (also known as the reference set). We set N to the number of annotated genes in the database from which we obtained the tested gene set. For the CAKUT causal genes set and the differentially expressed genes set, we used the largest N among the different databases, which corresponds to Gene Ontology. The null hypothesis is that the vitamin target genes are not associated with the gene set and the selection of k genes from that gene set is just a result of simple random sampling.

We performed a hypergeometric test for all the gene sets. A consequence of multiple testing is false discoveries in which null hypothesis is rejected erroneously. To control for the false discovery rate, we used the Benjamini-Hochberg correction method (BH adjusted).

### Supplementary analyses

We performed two supplementary analyses.

• We calculated the overlap statistics with a randomized sampling approach instead of hypergeometric test as a confirmation
• We calculated the overlaps of the CAKUT causal genes set with the other CAKUT-related gene sets (without considering being a vitamin target)

The details of the supplementary analyses and results are provided in *Extended data*. ^32^

## Results and discussion

The results of the overlap analyses between vitamin A and D target genes and CAKUT-related gene sets are presented in Tables 1 and 2, and the corresponding data is available in *Underlying data*. ^32^

**Table 1.** Overlap analysis results for vitamin A. Pub: Publications, ^3, 4, 33^ GO: Gene Ontology, Reac: Reactome, WP: WikiPathways.

Source	Term name	BH adjusted p-value and overlap with genes from Comparative Toxicogenomics Database	BH adjusted p-value and overlap with genes from Balmer and Blomhoff
Pub	CAKUT causal genes	(p-value = **1.90E-04**) BMP4, EYA1, GATA3, GREB1L, HNF1B, NRIP1, PBX1, RET, SLIT2, WNT4	(p-value = **2.35E-03**) AGTR1, BMP4, HNF1B, ITGA8, NRIP1, RET
Pub	CAKUT DEGs	(p-value = **1.39e-05**) CCL20, CCN2, DKK1, EEF1A2, GAD1, IL6, INA, LCN2, LGI1, MYC, NUSAP1, PTGS2, SGK1, SOCS2, TOP2A, TRIL	(p-value = 8.56e-02) IL6, KRT13, MYC, PTGS2, TOP2A
GO	Renal system development	(p-value = **3.62E-26**) ACTA2, ALDH1A2, ASS1, BAX, BCL2, BMP2, BMP4, BMP7, CASP9, CAT, CDKN1C, CFLAR, COL4A1, CTNNB1, CYP26A1, CYP26B1, DCN, EFNB2, EGR1, EYA1, FGF10, FGF2, FGF8, FOXC1, GATA3, GREB1L, HAS2, HES1, HNF1B, HOXB7, HPGD, ID2, ID3, IRX3, ITGA3, JAG1, LAMA5, LHX1, MMP9, MYC, NFIA, NOTCH1, NOTCH2, ODC1, PBX1, PDGFA, PDGFRA, PDGFRB, PLCE1, RARA, RARB, RBP4, RDH10, RET, RIDA, SDC1, SERPINF1, SHH, SLIT2, SMAD2, SMAD3, SOX9, STAT1, STRA6, SULF2, TACSTD2, TFAP2A, TGFB1, TGFB2, TGFBR1, TIPARP, VEGFA, WNT4, WNT5A, WT1, ZBTB16	(p-value = **6.44E-17**) ACTA2, AGTR1, BCL2, BMP2, BMP4, BMP7, CA2, CALB1, COL4A1, CTNNB1, EGR1, FGF1, FGF2, FGFR2, HNF1B, HOXD11, IL6R, ITGA8, LHX1, MME, MMP9, MYC, NOTCH1, ODC1, PDGFA, PDGFRA, PDGFRB, PECAM1, RARA, RARB, RBP4, RET, SHH, SOX9, STAT1, STRA6, TFAP2A, TGFB1, TGFB2, TGFBR1, VEGFA, WNT1, WT1
GO	Kidney development	(p-value = **4.21E-25**) ACTA2, ALDH1A2, ASS1, BAX, BCL2, BMP2, BMP4, BMP7, CASP9, CAT, CDKN1C, CFLAR, CTNNB1, CYP26A1, CYP26B1, DCN, EFNB2, EGR1, EYA1, FGF10, FGF2, FGF8, FOXC1, GATA3, GREB1L, HAS2, HES1, HNF1B, HOXB7, HPGD, ID2, ID3, IRX3, ITGA3, JAG1, LAMA5, LHX1, MMP9, MYC, NOTCH1, NOTCH2, ODC1, PBX1, PDGFA, PDGFRA, PDGFRB, PLCE1, RARA, RARB, RDH10, RET, RIDA, SDC1, SERPINF1, SHH, SLIT2, SMAD2, SMAD3, SOX9, STAT1, STRA6, SULF2, TACSTD2, TFAP2A, TGFB1, TGFB2, TGFBR1, TIPARP, VEGFA, WNT4, WNT5A, WT1, ZBTB16	(p-value = **3.90E-16**) ACTA2, AGTR1, BCL2, BMP2, BMP4, BMP7, CA2, CALB1, CTNNB1, EGR1, FGF1, FGF2, FGFR2, HNF1B, HOXD11, IL6R, ITGA8, LHX1, MME, MMP9, MYC, NOTCH1, ODC1, PDGFA, PDGFRA, PDGFRB, PECAM1, RARA, RARB, RET, SHH, SOX9, STAT1, STRA6, TFAP2A, TGFB1, TGFB2, TGFBR1, VEGFA, WNT1, WT1
GO	Kidney morphogenesis	(p-value = **2.17E-12**) BCL2, BMP2, BMP4, BMP7, CTNNB1, EYA1, FGF10, FGF2, FGF8, GATA3, GREB1L, HES1, HNF1B, HOXB7, IRX3, LAMA5, LHX1, MYC, PBX1, PDGFRB, SHH, SOX9, STAT1, TACSTD2, TGFB1, VEGFA, WNT4, WT1	(p-value = **7.49E-12**) BCL2, BMP2, BMP4, BMP7, CALB1, CTNNB1, FGF1, FGF2, HNF1B, HOXD11, LHX1, MYC, PDGFRB, SHH, SOX9, STAT1, TGFB1, VEGFA, WNT1, WT1
Reac	Metabolism of Angiotensinogen to Angiotensins	(p-value = 4.30E-01) CTSD, CTSG	(p-value = 6.41E-02) CTSD, CTSG, MME
Reac	RET signaling	(p-value = 1.82E-01) GRB10, IRS2, PIK3R1, PRKCA, RET	(p-value = 2.93E-01) GFRA1, PRKCA, RET
Reac	Signaling by WNT	(p-value = 1.46E-01) AKT1, CLTB, CTBP2, CTNNB1, DACT1, DKK1, FZD2, GNB4, GNG4, GSK3B, H2AC6, H2BC12, H4-16, ITPR3, MYC, PPP3CA, PRICKLE1, PRKCA, PRKCB, PSMB8, PSMB9, PSME2, RAC1, SOX3, SOX7, SOX9, TERT, WNT3, WNT4, WNT5A, XIAP	(p-value = 5.45E-01) CAMK2A, CLTA, CTNNB1, LEF1, MYC, PPP3CA, PPP3CB, PRKCA, RUNX3, SOX9, TERT, WNT1, WNT3A, WNT8A
Reac	Signaling by NOTCH	(p-value = **1.08E-03**) ACTA2, ADAM10, AKT1, CCND1, CREB1, DLK1, EGFR, ELF3, FABP7, H2AC6, H2BC12, H4-16, HES1, HEY1, JAG1, JUN, LFNG, MDK, MYC, NOTCH1, NOTCH2, PBX1, PSMB8, PSMB9, PSME2, RUNX1, SMAD3, ST3GAL4, ST3GAL6, STAT1, TFDP2, TP53	(p-value = 5.45E-01) ACTA2, CNTN1, EGFR, FCER2, JUN, MDK, MYC, NOTCH1, STAT1, TP53
Reac	Signaling by Hedgehog	(p-value = 8.84E-01) GSK3B, PSMB8, PSMB9, PSME2, SHH, TUBA4A, TUBB2B, TUBB3	(p-value = 9.87E-01) IHH, SHH
WP	Development of ureteric collection system	(p-value = **1.59E-05**) BMP4, CCND1, CTNNB1, FOXC1, GATA3, GREB1L, LHX1, MYCN, RARA, RARB, RARG, RET, SHH, SLIT2, TGFB2, WT1	(p-value = **7.21E-08**) BMP4, CTNNB1, FGFR2, GFRA1, HOXD11, ITGA8, LHX1, MYCN, RARA, RARB, RARG, RET, SHH, TGFB2, WT1
WP	Genes controlling nephrogenesis	(p-value = **1.84E-05**) CTNNB1, EYA1, FGF8, FOXC1, ITGA3, KDR, LHX1, NOTCH2, PDGFRB, RET, SHH, SLIT2, VEGFA, WNT4, WT1	(p-value = **2.92E-04**) CTNNB1, FGFR2, HOXD11, ITGA8, LHX1, PDGFRB, RET, SHH, VEGFA, WT1
WP	Nephrogenesis	(p-value = **1.90E-04**) ALDH1A2, BMP7, FGF8, GREB1L, JAG1, MEIS1, NOTCH2, WNT4	(p-value = 3.16E-01) BMP7, MEIS1
WP	GDNF/RET signaling axis	(p-value = **1.57E-03**) BMP4, CTNNB1, EYA1, FOXC1, GATA3, LHX1, RET, SLIT2	(p-value = **1.61E-02**) BMP4, CTNNB1, GFRA1, LHX1, RET

**Table 2.** Overlap analysis results for vitamin D. Pub: Publications, ^3, 4, 33^ GO: Gene Ontology, Reac: Reactome, WP: WikiPathways.

Source	Term name	BH adjusted p-value and overlap with genes from Comparative Toxicogenomics Database	BH adjusted p-value and overlap with genes from Ramagopalan *et al.*
Pub	CAKUT causal genes	(p-value = 1)	(p-value = 1)
Pub	CAKUT DEGs	(p-value = 6.84e-02) ANGPT2, GEM, MYC, PTGS2	(p-value = 7.64e-01) CD79A, IRF8
GO	Renal system development	(p-value = **1.45E-05**) ANGPT2, BAX, BCL2, CA2, CALB1, CD24, CYP26B1, DCN, EFNB2, GREM1, ID2, ITGA3, MYC, OSR2, SMAD7, TACSTD2, TGFB2	(p-value = 1) CFLAR, SULF2
GO	Kidney development	(p-value = **1.45E-05**) ANGPT2, BAX, BCL2, CA2, CALB1, CD24, CYP26B1, DCN, EFNB2, GREM1, ID2, ITGA3, MYC, OSR2, SMAD7, TACSTD2, TGFB2	(p-value = 1) CFLAR, SULF2
GO	Kidney morphogenesis	(p-value = 5.07E-02) BCL2, CALB1, GREM1, MYC, TACSTD2	(p-value = 1)
Reac	Metabolism of Angiotensinogen to Angiotensins	(p-value = 1)	(p-value = 7.64E-01) CTSZ
Reac	RET signaling	(p-value = 1)	(p-value = 7.64E-01) FRS2, PIK3CA
Reac	Signaling by WNT	(p-value = 1) ITPR1, MYC, XPO1	(p-value = 1) DAAM1, PIP5K1B, RAC2
Reac	Signaling by NOTCH	(p-value = 7.37E-01) CCND1, ELF3, HIF1A, LFNG, MYC, NCOR2	(p-value = 1) HIF1A, KAT2B, MAML1
Reac	Signaling by Hedgehog	(p-value = 1)	(p-value = 1)
WP	Development of ureteric collection system	(p-value = 7.39E-01) CCND1, TGFB2	(p-value = 1)
WP	Genes controlling nephrogenesis	(p-value = 1) ITGA3	(p-value = 7.64E-01) CD2AP, CXCR4
WP	Nephrogenesis	(p-value = 1)	(p-value = 1)
WP	GDNF/RET signaling axis	(p-value = 8.65E-01) GREM1	(p-value = 1)

### Vitamin A target genes and CAKUT-related gene sets show significant overlaps

Vitamin A target genes obtained from both CTD and Balmer and Blomhoff are significantly enriched in the set of CAKUT causal genes, with an overlap of ten and six genes, respectively ( Table 1). Vitamin A target genes obtained from CTD are also significantly enriched in the set of CAKUT differentially expressed genes (DEGs).

Significant overlaps are observed focusing on GO terms related to renal system development, kidney development, and kidney morphogenesis. These results are expected due to the recognized role of vitamin A in differentiation. ^46, 47^ It should be noted that all overlapping CAKUT causal genes except NRIP1 are also part of the kidney development GO term.

Four genes (BMP4, HNF1B, RET, NRIP1) appear as particularly interesting as they are CAKUT causal genes and vitamin A targets according to both CTD and Balmer and Blomhoff. BMP4 is a growth factor of the TGF-beta family involved in a wide variety of developmental processes. ^48^ BMP4 inhibits ectopic budding of the ureteric tips and promotes elongation of the ureter. ^49^ RET is a tyrosine-protein kinase receptor, involved in a large variety of cellular processes. RET signaling is important, in particular, for the terminal growth of the nephric duct, and for initial formation and outgrowth of the ureteric bud. ^50^ The Hepatocyte Nuclear Factor 1-beta (HNF1B) is a transcription factor involved in ureteric bud branching and induction of nephrogenesis. It is required for normal S-shaped bodies patterning and subsequent morphogenesis of all nephron segments. ^51^ Finally, Nuclear receptor interacting protein 1 (NRIP1) modulates the activity of nuclear receptors. It has been shown to play roles in renal malformations via deregulations of retinoic acid signaling. ^52^

Concerning the overlap with Reactome pathways, the vitamin A target genes obtained from CTD are significantly enriched in signaling by NOTCH. The NOTCH signaling pathway is mainly involved in cell-to-cell communication, ^53^ and it plays a role in the development of most organs and tissues. ^54^ Other Reactome terms also have overlapping genes, even if not significant. This includes the WNT signaling pathway (which is involved in developmental processes such as patterning, differentiation/proliferation shift, and migration) and the metabolism of angiotensinogen to angiotensins pathway, from which CTSD, CTSG and MME are vitamin A targets and play roles in the generation of different angiotensin peptides. ^55, 56, 57^ The impaired conversion of angiotensin I to angiotensin II is the pharmacodynamic adverse mechanism underlying the fetopathy caused by ACE-inhibiting drugs. ^58^ One main feature of this teratogen-induced fetopathy, namely renal tubular dysgenesis, belongs to the CAKUT spectrum. In addition, an overproduction of angiotensin II might have a role in CAKUT anomalies involving renal tubules, as observed in cases of the autosomal recessive polycystic kidney disease. ^59^

Finally, vitamin A target genes extracted from both CTD and Balmer and Blomhoff are significantly enriched in the development of ureteric collection system, genes controlling nephrogenesis, and GDNF/RET signaling axis pathways, as defined in WikiPathways. The target genes extracted from CTD are also significantly enriched in nephrogenesis. Signaling by GDNF through the RET receptor is required for normal growth of the ureteric bud. ^60^ Localized expression of GDNF by the metanephric mesenchyme is important to elicit and correctly position the initial budding event from the Wolffian duct and to promote the continued branching of the ureteric bud.

Overall, the significant overlaps observed between vitamin A and the CAKUT gene sets indicate that vitamin A might be relevant to a broad range of urogenital abnormalities.

### Vitamin D target genes overlap with renal system development

In the analyses of vitamin D target gene sets, we overall observed very few significant overlaps with CAKUT-related gene sets. Vitamin D target genes obtained from CTD are only significantly enriched in renal system development and kidney development GO terms. It should be noted that in each case 70% of the enriched genes are also vitamin A target genes (according to CTD or Balmer and Blomhoff).

CTSZ, which cleaves angiotensin I to yield angiotensin II, ^61^ is the only vitamin D target in the metabolism angiotensinogen to angiotensins pathway. Thus, within CAKUT, vitamin D might also have a role in renal tubular defects related to anomalies of the renin-angiotensin system.

WNT and NOTCH signaling pathways have genes that are targets of vitamin D but there is a small, non-significant overlap, and the overlapping genes are not the WNT and NOTCH receptors nor ligands.

## Conclusions

In this study we examined the overlaps of vitamin A and vitamin D target gene sets extracted from different databases and publications with CAKUT-related genes and biological processes. While we only observed significant enrichment of vitamin D target genes in GO kidney development terms, there is significant enrichment of vitamin A target genes in most of the gene sets we tested. Indeed, we observed that vitamin A target genes are enriched in CAKUT causal genes set, CAKUT differentially expressed genes set, kidney development pathways, the signaling by NOTCH pathway, and the GDNF/RET signaling axis pathway. There is also an overlap with the signaling by the WNT pathway, although it is not statistically significant. Overall, the significant overlaps observed between vitamin A and CAKUT gene sets indicate that vitamin A might be relevant to a broad range of urogenital abnormalities, pointing out the importance of nutritional management during pregnancy for fetal development. However, it should be clear that our study is dedicated to the generation of hypotheses. Additional studies are needed, using up-to-date datasets obtained with new omics techniques, to investigate the fine-grained effects of vitamin excess and deficiency on CAKUT, and to decipher the pathophysiological mechanisms. The increased availability of multi-omics technologies, such as whole genome sequencing and metabolomics will certainly open new directions, e.g. by revealing new causal genes or identifying metabolic disturbances due to environmental factors.

## Data availability

### Underlying data

Zenodo: Overlap of vitamin A and vitamin D target genes with CAKUT-related processes. https://www.doi.org/10.5281/zenodo.4501623. ^32^

This project contains the following underlying data in the ‘Data’ folder:

• VitA-CTD-Genes.txt (list of vitamin A target genes from CTD used for overlap analyses).
• VitA-Balmer2002-Genes.txt (list of vitamin A target genes from Balmer and Blomhoff used for overlap analyses).
• VitD-CTD-Genes.txt (list of vitamin D target genes from CTD used for overlap analyses).
• VitD-Ramagopalan2010.txt (list of vitamin D target genes from Ramagopalan *et al.* used for overlap analyses).
• PathwaysOfInterest.gmt (CAKUT-related gene sets used for overlap analyses).
• PathwaysOfInterestBackground.txt (list indicating which background GMT file to be used for the analysis of each CAKUT-related gene set).
• hsapiens.GO:BP.name.gmt (all gene sets from GO:BP used as background information for overlap analyses).
• hsapiens.REAC.name.gmt (all gene sets from Reactome used as background information for overlap analyses).
• hsapiens.WP.name.gmt (all gene sets from WikiPathways used as background information for overlap analyses).

This project contains the following underlying data in the ‘Result’ folder:

• VitA-CTD-Genes.csv (overlap analysis results for vitamin A targets from CTD).
• VitA-Balmer2002-Genes.csv (overlap analysis results for vitamin A targets from Balmer and Blomhoff).
• VitA-Merged.csv (merged table for the results presented in VitA-CTD-Genes.csv and VitA-Balmer2002-Genes.csv).
• VitD-CTD-Genes.csv (overlap analysis results for vitamin D targets from CTD).
• VitD-Ramagopalan2010.csv (overlap analysis results for vitamin D targets from Ramagopalan *et al.*).
• VitD-Merged.csv (merged table for the results presented in VitD-CTD-Genes.csv and VitD-Ramagopalan2010.csv).

### Extended data

Zenodo: Overlap of vitamin A and vitamin D target genes with CAKUT-related processes. https://www.doi.org/10.5281/zenodo.4501623. ^32^

This project contains the following extended data:

• overlapAnalysis.py (Python script used for all the analyses).

This project contains the following extended data in the ‘Supp’ folder:

• CAKUT-CausalGenesPMID29079659-PMID30143558.txt (list of genes known to be mutated in the monogenic form of CAKUT).
• DEGsFromPMID30261159.txt (list of differentially expressed genes in CAKUT)
• SupplementaryAnalyses.odt (descriptions of the supplementary analyses).
• targetsFromDifferentSourcesOverlap.odt (contains the information on vitamin A and D targets from different sources (CTD, publications), presents source specific genes and common genes).
• VitA-Balmer2002-GeneMapping.csv (detailed table for the gene list from Balmer and Blomhoff).

This project contains the following extended data in the ‘Supp/CAKUTCausalGenesOverlapWithOthers’ folder:

• CAKUTCausalGenesOverlap.csv (overlap of CAKUT causal genes with other CAKUT-related gene sets without considering whether genes are vitamin targets or not).

This project contains the following extended data in the ‘Supp/RandomizedSampling’ folder:

• VitA-CTD-Genes-RS.csv (overlap analysis results for vitamin A targets from CTD using the randomized sampling approach)
• VitA-Balmer2002-Genes-RS.csv (overlap analysis results for vitamin A targets from Balmer and Blomhoff using the randomized sampling approach - please see answer to the reviewers).
• VitD-CTD-Genes-RS.csv (overlap analysis results for vitamin D targets from CTD using the randomized sampling approach).
• VitD-Ramagopalan2010-RS.csv (overlap analysis results for vitamin D targets from Ramagopalan et al. using the randomized sampling approach)

Data are available under the terms of the Creative Commons Attribution 4.0 International license (CC-BY 4.0).

## Author contributions

Ozisik O: Conceptualization, Data Curation, Formal Analysis, Project Administration, Writing – Original Draft Preparation; Ehrhart F: Conceptualization, Writing – Review & Editing; Evelo C: Conceptualization, Supervision, Writing – Review & Editing; Mantovani A: Conceptualization, Supervision, Writing – Original Draft Preparation; Baudot A: Conceptualization, Data Curation, Project Administration, Supervision, Writing – Original Draft Preparation.

## References

[1] EU RD Platform prevalence charts and tables:2020. Accessed: 22.01.2021. Reference Source

[2] Nicolaou N Renkema KY Bongers EM : Genetic, environmental, and epigenetic factors involved in CAKUT. *Nat Rev Nephrol.* Dec 2015;11(12):720–731. 10.1038/nrneph.2015.140 26281895

[3] van der Ven AT Vivante A Hildebrandt F : Novel Insights into the Pathogenesis of Monogenic Congenital Anomalies of the Kidney and Urinary Tract. *J Am Soc Nephrol.* 01 2018;29(1):36–50. 10.1681/ASN.2017050561 29079659 PMC5748921

[4] van der Ven AT Connaughton DM Ityel H : Whole-Exome Sequencing Identifies Causative Mutations in Families with Congenital Anomalies of the Kidney and Urinary Tract. *J Am Soc Nephrol.* 09 2018;29(9):2348–2361. 10.1681/ASN.2017121265 30143558 PMC6115658

[5] Murugapoopathy V Gupta IR : A Primer on Congenital Anomalies of the Kidneys and Urinary Tracts (CAKUT). *Clin J Am Soc Nephrol.* 05 2020;15(5):723–731. 10.2215/CJN.12581019 32188635 PMC7269211

[6] Boubred F Vendemmia M Garcia-Meric P : Effects of maternally administered drugs on the fetal and neonatal kidney. *Drug Saf.* 2006;29(5):397–419. 10.2165/00002018-200629050-00004 16689556

[7] Parikh CR McCall D Engelman C : Congenital renal agenesis: case-control analysis of birth characteristics. *Am J Kidney Dis.* Apr 2002;39(4):689–694. 10.1053/ajkd.2002.31982 11920333

[8] Hsu CW Yamamoto KT Henry RK : Prenatal risk factors for childhood CKD. *J Am Soc Nephrol.* Sep 2014;25(9):2105–2111. 10.1681/ASN.2013060582 24744441 PMC4147970

[9] Dart AB Ruth CA Sellers EA : Maternal diabetes mellitus and congenital anomalies of the kidney and urinary tract (CAKUT) in the child. *Am J Kidney Dis.* May2015;65(5):684–691. 10.1053/j.ajkd.2014.11.017 25595566

[10] Tain YL Luh H Lin CY : Incidence and Risks of Congenital Anomalies of Kidney and Urinary Tract in Newborns: A Population-Based Case-Control Study in Taiwan. *Medicine (Baltimore).* Feb 2016;95(5):e2659. 10.1097/MD.0000000000002659 26844492 PMC4748909

[11] Groen In ’t Woud S Renkema KY Schreuder MF : Maternal risk factors involved in specific congenital anomalies of the kidney and urinary tract: A case-control study. *Birth Defects Res A Clin Mol Teratol.* Jul 2016;106(7):596–603. 10.1002/bdra.23500 27040999

[12] Postoev VA Grjibovski AM Kovalenko AA : Congenital anomalies of the kidney and the urinary tract: A murmansk county birth registry study. *Birth Defects Res A Clin Mol Teratol.* Mar 2016;106(3):185–193. 10.1002/bdra.23475 26833755

[13] Jadresić L Au H Woodhouse C : Pre-pregnancy obesity and risk of congenital abnormalities of the kidney and urinary tract (CAKUT)-systematic review, meta-analysis and ecological study. *Pediatr Nephrol.* Jan 2021;36(1):119–132. 10.1007/s00467-020-04679-0 32596798

[14] de Souza Mesquita LM Mennitti LV de Rosso VV : The role of vitamin A and its pro-vitamin carotenoids in fetal and neonatal programming: gaps in knowledge and metabolic pathways. *Nutr Rev.* Jan 2021;79(1):76–87. 10.1093/nutrit/nuaa075 33301001

[15] Burrow CR : Retinoids and renal development. *Exp Nephrol.* 2000;8(4-5):219–225. 10.1159/000020672 10940720

[16] Charlton JR Springsteen CH Carmody JB : Nephron number and its determinants in early life: a primer. *Pediatr Nephrol.* Dec 2014;29(12):2299–2308. 10.1007/s00467-014-2758-y 24488483

[17] Lee YQ Collins CE Gordon A : The Relationship between Maternal Nutrition during Pregnancy and Offspring Kidney Structure and Function in Humans: A Systematic Review. *Nutrients.* Feb 2018;10(2). 10.3390/nu10020241 29466283 PMC5852817

[18] Lumbers ER Kandasamy Y Delforce SJ : Programming of Renal Development and Chronic Disease in Adult Life. *Front Physiol.* 2020;11:757. 10.3389/fphys.2020.00757 32765290 PMC7378775

[19] Lelièvre-Pégorier M Vilar J Ferrier ML : Mild vitamin A deficiency leads to inborn nephron deficit in the rat. *Kidney Int.* Nov 1998;54(5):1455–1462. 10.1046/j.1523-1755.1998.00151.x 9844121

[20] Mendelsohn C Batourina E Fung S : Stromal cells mediate retinoid-dependent functions essential for renal development. *Development.* Mar 1999;126(6):1139–1148. 10021334 10.1242/dev.126.6.1139

[21] Batourina E Gim S Bello N : Vitamin A controls epithelial/mesenchymal interactions through Ret expression. *Nat Genet.* Jan 2001;27(1):74–78. 10.1038/83792 11138002

[22] Goodyer P Kurpad A Rekha S : Effects of maternal vitamin A status on kidney development: a pilot study. *Pediatr Nephrol.* Feb 2007;22(2):209–214. 10.1007/s00467-006-0213-4 17093988

[23] El-Khashab EK Hamdy AM Maher KM : Effect of maternal vitamin A deficiency during pregnancy on neonatal kidney size. *J Perinat Med.* Mar 2013;41(2):199–203. 10.1515/jpm-2012-0026 23093302

[24] Rogers SA Droege D Dusso A : Incubation of metanephroi with vitamin d(3) increases numbers of glomeruli. *Organogenesis.* Oct 2004;1(2):52–54. 10.4161/org.1.2.1292 19521561 PMC2633986

[25] Maka N Makrakis J Parkington HC : Vitamin D deficiency during pregnancy and lactation stimulates nephrogenesis in rat offspring. *Pediatr Nephrol.* Jan 2008;23(1):55–61. 10.1007/s00467-007-0641-9 17965890

[26] Nascimento FA Ceciliano TC Aguila MB : Maternal vitamin D deficiency delays glomerular maturity in F1 and F2 offspring. *PLoS One.* 2012;7(8):e41740. 10.1371/journal.pone.0041740 22927914 PMC3424155

[27] Boyce AC Palmer-Aronsten BJ Kim MY : Maternal vitamin D deficiency programmes adult renal renin gene expression and renal function. *J Dev Orig Health Dis.* Oct 2013;4(5):368–376. 10.1017/S2040174413000342 24970730

[28] Miliku K Voortman T Franco OH : Vitamin D status during fetal life and childhood kidney outcomes. *Eur J Clin Nutr.* 05 2016;70(5):629–634. 10.1038/ejcn.2015.216 26695721 PMC5576536

[29] Davis AP Grondin CJ Johnson RJ : Comparative Toxicogenomics Database (CTD): update 2021. Nucleic Acids Res. Jan 2021;49(D1):D1138–D1143. 10.1093/nar/gkaa891 33068428 PMC7779006

[30] Balmer JE Blomhoff R : Gene expression regulation by retinoic acid. *J Lipid Res.* Nov 2002;43(11):1773–1808. 10.1194/jlr.r100015-jlr200 12401878

[31] Ramagopalan SV Heger A Berlanga AJ : A ChIP-seq defined genome-wide map of vitamin D receptor binding: associations with disease and evolution. *Genome Res.* Oct 2010;20(10):1352–1360. 10.1101/gr.107920.110 20736230 PMC2945184

[32] Ozisik O Ehrhart F Evelo C : Overlap of vitamin A and vitamin D target genes with CAKUT-related processes. Zenodo. April 2022. 10.5281/zenodo.4501623 PMC9051587 35528959

[33] Jovanovic I Zivkovic M Kostic M : Transcriptome-driven integrative exploration of functional state of ureter tissue affected by CAKUT. Life Sci. Nov 2018;212:1–8. 10.1016/j.lfs.2018.09.042 30261159

[34] Ashburner M Ball CA Blake JA : Gene ontology: tool for the unification of biology. The Gene Ontology Consortium. *Nat Genet.* May 2000;25(1):25–29. 10.1038/75556 10802651 PMC3037419

[35] The Gene Ontology Consortium: The Gene Ontology Resource: 20 years and still GOing strong. *Nucleic Acids Res.* 01 2019;47(D1):D330–D338. 10.1093/nar/gky1055 30395331 PMC6323945

[36] Yosypiv IV : Renin-angiotensin system in mammalian kidney development. *Pediatr Nephrol.* Feb 2020. 10.1007/s00467-020-04496-5 32072306

[37] Davis TK Hoshi M Jain S : To bud or not to bud: the RET perspective in CAKUT. *Pediatr Nephrol.* Apr 2014;29(4):597–608. 10.1007/s00467-013-2606-5 24022366 PMC3952039

[38] Kreidberg JA : Murine models of kidney development. In: Pollak MR Mount DB , editor, *Molecular and Genetic Basis of Renal Disease.* 2014chapter 4, pages41–48. W.B. Saunders.

[39] Pulkkinen K Murugan S Vainio S : Wnt signaling in kidney development and disease. *Organogenesis.* Apr 2008;4(2):55–59. 10.4161/org.4.2.5849 19279716 PMC2634248

[40] Sirin Y Susztak K : Notch in the kidney: development and disease. *J Pathol.* Jan 2012;226(2):394–403. 10.1002/path.2967 21952830 PMC3677191

[41] Edeling M Ragi G Huang S : Developmental signalling pathways in renal fibrosis: the roles of Notch, Wnt and Hedgehog. *Nat Rev Nephrol.* 07 2016;12(7):426–439. 10.1038/nrneph.2016.54 27140856 PMC5529143

[42] Wang Y Zhou CJ Liu Y : Wnt Signaling in Kidney Development and Disease. *Prog Mol Biol Transl Sci.* 01 2018;153:181–207. 10.1016/bs.pmbts.2017.11.019 29389516 PMC6008255

[43] Mukherjee M Fogarty E Janga M : Notch Signaling in Kidney Development, Maintenance, and Disease. *Biomolecules.* 11 2019;9(11). 10.3390/biom9110692 31690016 PMC6920979

[44] Jassal B Matthews L Viteri G : The reactome pathway knowledgebase. *Nucleic Acids Res.* 01 2020;48(D1):D498–D503. 10.1093/nar/gkz1031 31691815 PMC7145712

[45] Martens M Ammar A Riutta A : WikiPathways: connecting communities. *Nucleic Acids Res.* 01 2021;49(D1):D613–D621. 10.1093/nar/gkaa1024 33211851 PMC7779061

[46] Ross AC Gardner EM : The function of vitamin A in cellular growth and differentiation, and its roles during pregnancy and lactation. *Adv Exp Med Biol.* 1994;352:187–200. 10.1007/978-1-4899-2575-6_15 7832047

[47] Love JM Gudas LJ : Vitamin A, differentiation and cancer. *Curr Opin Cell Biol.* Dec 1994;6(6):825–831. 10.1016/0955-0674(94)90051-5 7880529

[48] Rezzola S Di Somma M Corsini M : VEGFR2 activation mediates the pro-angiogenic activity of BMP4. Angiogenesis. Nov 2019;22(4):521–533. 10.1007/s10456-019-09676-y 31363885

[49] Miyazaki Y Oshima K Fogo A : Bone morphogenetic protein 4 regulates the budding site and elongation of the mouse ureter. J Clin Invest. Apr 2000;105(7):863–873. 10.1172/JCI8256 10749566 PMC377476

[50] Costantini F : RET signaling in ureteric bud formation and branching. In: Little MH , editor, Kidney Development, Disease, Repair and Regeneration.chapter 4, pp.41–56. Academic Press;2016. 10.1016/B978-0-12-800102-8.00004-7

[51] Heliot C Desgrange A Buisson I : HNF1B controls proximal-intermediate nephron segment identity in vertebrates by regulating Notch signalling components and Irx1/2. Development. Feb 2013;140(4):873–885. 10.1242/dev.086538 23362348

[52] Vivante A Mann N Yonath H : Dysregulation of Retinoic Acid Signaling. J Am Soc Nephrol. Aug 2017;28(8):2364–2376. 10.1681/ASN.2016060694 28381549 PMC5533226

[53] Bigas A Espinosa L : Notch signaling in cell–cell communication pathways. *Current Stem Cell Reports.* Dec 2016;2(4):349–355. 10.1007/s40778-016-0065-1

[54] Siebel C Lendahl U : Notch Signaling in Development, Tissue Homeostasis, and Disease. *Physiol Rev.* 10 2017;97(4):1235–1294. 10.1152/physrev.00005.2017 28794168

[55] Belova LA : Angiotensin II-generating enzymes. *Biochemistry (Moscow).* Dec 2000;65(12):1337–1345. 10.1023/a:1002848402911 11173502

[56] Rice GI Thomas DA Grant PJ : Evaluation of angiotensin-converting enzyme (ACE), its homologue ACE2 and neprilysin in angiotensin peptide metabolism. *Biochem J.* Oct 2004;383(Pt 1):45–51. 10.1042/BJ20040634 15283675 PMC1134042

[57] Wu C Lu H Cassis LA : Molecular and Pathophysiological Features of Angiotensinogen: A Mini Review. *N Am J Med Sci (Boston).* Oct 2011;4(4):183–190. 22389749 10.7156/v4i4p183 PMC3291105

[58] Buttar HS : An overview of the influence of ACE inhibitors on fetal-placental circulation and perinatal development. *Mol Cell Biochem.* Nov 1997;176(1-2):61–71. 9406146

[59] Loghman-Adham M Soto CE Inagami T : Expression of components of the renin-angiotensin system in autosomal recessive polycystic kidney disease. *J Histochem Cytochem.* Aug 2005;53(8):979–988. 10.1369/jhc.4A6494.2005 15879580

[60] Costantini F Shakya R : GDNF/Ret signaling and the development of the kidney. *Bioessays.* Feb 2006;28(2):117–127. 10.1002/bies.20357 16435290

[61] Nägler DK Kraus S Feierler J : A cysteine-type carboxypeptidase, cathepsin X, generates peptide receptor agonists. *Int Immunopharmacol.* Jan 2010;10(1):134–139. 10.1016/j.intimp.2009.09.018 19800993

