# Supplementary material for: Overlap of vitamin A and vitamin D target genes with CAKUT-related processes
Source: F1000Res. 2022 Apr 21;10:395. Originally published 2021 May 18. [Version 2] doi: 10.12688/f1000research.51018.2 (PMC9051587; doi:10.12688/f1000research.51018.2)
Supplement: Peer review history

# Peer review: Reviewer response for version 2

**DOI:** 10.5256/f1000research.132373.r135410

**Published:** 2022-05-03

**Authors:** Elena Menegola

**Affiliations:** 1 Department of Environmental Sciences and Policies, Università degli Studi di Milano, Milan, Italy

After checking the revised form of the manuscript, my suggestion is Approved.

Is the work clearly and accurately presented and does it cite the current literature?

Partly

If applicable, is the statistical analysis and its interpretation appropriate?

I cannot comment. A qualified statistician is required.

Are all the source data underlying the results available to ensure full reproducibility?

Yes

Is the study design appropriate and is the work technically sound?

Yes

Are the conclusions drawn adequately supported by the results?

Partly

Are sufficient details of methods and analysis provided to allow replication by others?

Yes

Reviewer Expertise:

Developmental toxicology

I confirm that I have read this submission and believe that I have an appropriate level of expertise to confirm that it is of an acceptable scientific standard.

# Peer review: Reviewer response for version 2

**DOI:** 10.5256/f1000research.132373.r135411

**Published:** 2022-04-28

**Authors:** Olivia Angelin-Bonnet

**Affiliations:** 1 School of Fundamental Sciences, College of Sciences, Massey University, Palmerston North, New Zealand

The authors answered satisfactorily my comments. The added paragraph in the Methods section about the hypergeometric is clear and will be helpful to readers.

Is the work clearly and accurately presented and does it cite the current literature?

Yes

If applicable, is the statistical analysis and its interpretation appropriate?

Yes

Are all the source data underlying the results available to ensure full reproducibility?

Yes

Is the study design appropriate and is the work technically sound?

Yes

Are the conclusions drawn adequately supported by the results?

Yes

Are sufficient details of methods and analysis provided to allow replication by others?

Yes

Reviewer Expertise:

Statistics, Systems Biology, Biological networks analysis and reconstruction

I confirm that I have read this submission and believe that I have an appropriate level of expertise to confirm that it is of an acceptable scientific standard.

# Peer review: Reviewer response for version 1

**DOI:** 10.5256/f1000research.54123.r100885

**Published:** 2021-12-17

**Authors:** Sean Eddy

**Affiliations:** 1 Division of Nephrology, Michigan Medicine, University of Michigan, Ann Arbor, MI, USA

This study involves generating curated vitamin A and D gene sets from the public domain and assessing the relevance of the genes to CAKUT and CAKUT related pathways. The authors meticulously report their results and curation efforts so the findings are readily reproducible and are appropriate for F1000Research. 

*Is the study design appropriate and is the work technically sound?*

 The addition of targeted vitamin A/retinoic acid and D gene sets is appreciated. Limiting CTD exported gene sets to those with at least 2 references is a reasonable approach to reducing potential false positives. I agree with the authors that this study is currently hypothesis generating. Given that patient CAKUT gene sets already exist and are publicly available (Jovanich *et al*., 2018, GEO dataset GSE **GSE83946**) ^1^, the authors should also assess the enrichment of their gene sets against differentially expressed genes from patients with CAKUT and healthy control reference tissue.

*If applicable, is the statistical analysis and its interpretation appropriate?*

 Because the gene sets are substantially different in size, a direct comparison of enrichment and p-values can be misleading. I would suggest the authors generate a randomized gene set of equivalent size to each of the vitamin A and vitamin D gene sets and perform comparative enrichment analyses on the relevant pathways. 

 While not necessary, the addition of an odds ratio from 2x2 contingency matrices to assess the strength of enrichment would enhance the findings. 

 The targeted enrichment analysis of kidney and CAKUT related pathways is a reasonable approach to demonstrating a positive finding, ie. the gene sets identified associated with some of the expected/hypothesized pathways, it would be interesting to see if a more unbiased enrichment approach might also highlight other vitamin A and D relevant pathways, and perhaps additional renal-associated pathways not readily apparent from the targeted approach. This which could also substantiate the authors' vitamin A and D curation efforts.

Is the work clearly and accurately presented and does it cite the current literature?

Yes

If applicable, is the statistical analysis and its interpretation appropriate?

Partly

Are all the source data underlying the results available to ensure full reproducibility?

Yes

Is the study design appropriate and is the work technically sound?

Partly

Are the conclusions drawn adequately supported by the results?

Yes

Are sufficient details of methods and analysis provided to allow replication by others?

Yes

Reviewer Expertise:

Renal systems and computational biology.

I confirm that I have read this submission and believe that I have an appropriate level of expertise to confirm that it is of an acceptable scientific standard, however I have significant reservations, as outlined above.

## References

[1] : Transcriptome-driven integrative exploration of functional state of ureter tissue affected by CAKUT. *Life Sci* .2018;212: 10.1016/j.lfs.2018.09.042 1-8 10.1016/j.lfs.2018.09.042 30261159

# Response

**Published:** 2022-04-15

**Authors:** Ozan Ozisik

**Affiliations:** Aix Marseille University, France

We thank the reviewer for the valuable comments. We present the reviewer comments, our answers to them, and quoted texts from the revised manuscript below.

**Given that patient CAKUT gene sets already exist and are publicly available (Jovanich *et al*., 2018, GEO dataset GSE** GSE83946 **)** ^1^ **, the authors should also assess the enrichment of their gene sets against differentially expressed genes from patients with CAKUT and healthy control reference tissue.**

 We performed the suggested analysis, described the analysis in the manuscript and presented the results. We observed that vitamin A targets obtained from CTD significantly overlapped with the differentially expressed genes.

*“CAKUT differentially expressed genes set*

*Jovanovic et al. ^33^ identified 27 upregulated and 51 downregulated genes as a result of the transcriptome profiling of 15 CAKUT and 7 control ureter samples. We retrieved these differentially expressed genes (DEGs) from the supplementary file they provided. We updated gene names with the help of HUGO Gene Nomenclature Committee (HGNC) and obtained a final list of 74 DEGs (see Extended data ^32^ ).”*

**Because the gene sets are substantially different in size, a direct comparison of enrichment and p-values can be misleading. I would suggest the authors generate a randomized gene set of equivalent size to each of the vitamin A and vitamin D gene sets and perform comparative enrichment analyses on the relevant pathways.**

 We used hypergeometric tests in order to take into account the sizes of gene sets. Indeed, these tests are used in enrichment analyses to assess sets of genes’ overrepresentation in annotation terms of different sizes. However, we do agree that the experiment proposed by the reviewer is interesting to confirm our observations. We hence additionally performed a randomized sampling analysis.

 We selected a set of random genes having the same size as the target genes set from the background genes list. We repeated this selection 2000 times. We counted the number of times that the random set had better overlap with the CAKUT-related gene set than the target genes set.

 The results are consistent with the hypergeometric tests, except for two comparisons in which the overlaps were not significant according to the hypergeometric test but are significant according to the randomized sampling approach. Indeed, the adjusted p-value for the overlap between vitamin A targets retrieved from CTD and Reactome Signaling by WNT term is 0.146 (>0.05, insignificant) in the hypergeometric test but it is 0.0426 (<0.05, significant) in randomized sampling. Additionally, the adjusted p-value for the overlap between vitamin D targets retrieved from CTD and GO kidney morphogenesis term is 0.0507 (>0.05, insignificant) in the hypergeometric test but it is 0.042 (<0.05, significant) in randomized sampling. We should note that the results of the randomized sampling approach may change in different runs.

 As this is an extra test for confirmation, we provided related documents in Underlying data and Extended data for the interested readers. The analysis process is described in “Supp/SupplementaryAnalyses.odt”. The code of the randomized sampling test is available as part of overlapAnalysis.py; the results are in the “Supp/RandomizedSampling” folder.

**While not necessary, the addition of an odds ratio from 2x2 contingency matrices to assess the strength of enrichment would enhance the findings.**

 All the numbers necessary to compute the enrichments (gene set sizes, domain sizes, intersection sizes, p-values) are provided in the tables. We think that providing additional tables and metrics might be confusing.

**The targeted enrichment analysis of kidney and CAKUT related pathways is a reasonable approach to demonstrating a positive finding, ie. the gene sets identified associated with some of the expected/hypothesized pathways, it would be interesting to see if a more unbiased enrichment approach might also highlight other vitamin A and D relevant pathways, and perhaps additional renal-associated pathways not readily apparent from the targeted approach. This which could also substantiate the authors' vitamin A and D curation efforts.**

 We agree that our initial analyses could be extended. As a matter of fact, we are currently extending our approach from CAKUT-associated pathways to the available rare disease pathways. However providing all the enrichment results for all the pathways without any interpretation will not be useful for the audience. As we provide the vitamin target gene lists and the code to reproduce or extend our work, the interested (and expert) researchers with a precise question in mind could perform the analyses.

# Peer review: Reviewer response for version 1

**DOI:** 10.5256/f1000research.54123.r98474

**Published:** 2021-12-15

**Authors:** Olivia Angelin-Bonnet

**Affiliations:** 1 School of Fundamental Sciences, College of Sciences, Massey University, Palmerston North, New Zealand

The article "Overlap of vitamin A and vitamin D target genes with CAKUT-related processes" investigates the overlap between vitamin A and D target genes and genes associated with congenital anomalies of the kidney and urinary tract (CAKUT). More specifically, the authors assessed the significance of the intersection between sets of genes identified by previous publications or through databases as targets of either vitamin A or D, and sets of genes identified as causal to CAKUT or associated with pathways of interest with respect to CAKUT. This review focuses on the statistical analysis presented in this paper.

 I find the use of a hypergeometric test for this analysis completely appropriate. However, I found that while the Methods section provides a good explanation of the construction of the different gene sets investigated, there is no presentation or discussion of the statistical test used to assess the significance of the overlap between different gene sets. More specifically, I would like to see a brief explanation of the hypergeometric test, in order to justify its use to the user, as well as a statement of its null hypothesis. In addition, there is no mention of the background sets used for the test. I think they should be explicitly mentioned, especially since they differ with the pathway of interest tested (e.g. all GO terms for GO-related gene sets, all Reactome genes for Reactome sets, etc) and are used to constrain the sets of vitamin-related genes (i.e. how many vitamin genes were discarded because they did not appear in the background set?). I also want to note that the availability of the data and the (clearly commented) python script was really appreciated. It ensures that the statistical analysis can be fully reproduced.

 Lastly, a minor presentation point: I am not sure that having the p-value between brackets in the same cell as the list of interesting genes in the two tables is very clear, especially when the intersection set is empty and the resulting p-value is 1. I would suggest to add "p-value = " before the actual value to make it clearer.

Is the work clearly and accurately presented and does it cite the current literature?

Yes

If applicable, is the statistical analysis and its interpretation appropriate?

Yes

Are all the source data underlying the results available to ensure full reproducibility?

Yes

Is the study design appropriate and is the work technically sound?

Yes

Are the conclusions drawn adequately supported by the results?

Yes

Are sufficient details of methods and analysis provided to allow replication by others?

Yes

Reviewer Expertise:

Statistics, Systems Biology, Biological networks analysis and reconstruction

I confirm that I have read this submission and believe that I have an appropriate level of expertise to confirm that it is of an acceptable scientific standard, however I have significant reservations, as outlined above.

# Response

**Published:** 2022-04-15

**Authors:** Ozan Ozisik

**Affiliations:** Aix Marseille University, France

We thank the reviewer for the valuable comments. We present the reviewer comments, our answers to them, and quoted texts from the revised manuscript below.

**I find the use of a hypergeometric test for this analysis completely appropriate. However, I found that while the Methods section provides a good explanation of the construction of the different gene sets investigated, there is no presentation or discussion of the statistical test used to assess the significance of the overlap between different gene sets. More specifically, I would like to see a brief explanation of the hypergeometric test, in order to justify its use to the user, as well as a statement of its null hypothesis. In addition, there is no mention of the background sets used for the test. I think they should be explicitly mentioned, especially since they differ with the pathway of interest tested (e.g. all GO terms for GO-related gene sets, all Reactome genes for Reactome sets, etc) and are used to constrain the sets of vitamin-related genes (i.e. how many vitamin genes were discarded because they did not appear in the background set?). I also want to note that the availability of the data and the (clearly commented) python script was really appreciated. It ensures that the statistical analysis can be fully reproduced.**

 We thank the reviewer for pointing out the lack of necessary information on the applied test. We added the explanations for the statistical test, the null hypothesis, and the background sets used in the tests to the “Methods” section under “Overlap analyses” heading.

*“We computed the overlaps between vitamin target gene sets and the CAKUT gene sets of interest defined previously. After obtaining the overlap results, we calculated the significance of the overlaps using the hypergeometric test . Hypergeometric test calculates the statistical significance of getting k successes in n draws without replacement from a finite population of size N that contains a total of K successes. In our context, k is the number of genes overlapping with a specific gene set, K is the size of that gene set, n is the number of vitamin targets, and N is the background gene set (also known as the reference set). We set N to the number of annotated genes in the database from which we obtained the tested gene set. For the CAKUT causal genes set and the differentially expressed genes set, we used the largest N among the different databases, which corresponds to Gene Ontology. The null hypothesis is that the vitamin target genes are not associated with the gene set and the selection of k genes from that gene set is just a result of simple random sampling.*

*We performed a hypergeometric test for all the gene sets. A consequence of multiple testing is false discoveries in which null hypothesis is rejected erroneously. To control for the false discovery rate, we used the Benjamini-Hochberg correction method (BH adjusted).”*

**I would suggest to add "p-value = " before the actual value to make it clearer.**

 As suggested, we added “p-value = ” before the actual value.

# Peer review: Reviewer response for version 1

**DOI:** 10.5256/f1000research.54123.r95476

**Published:** 2021-10-27

**Authors:** Matias Simons

**Affiliations:** 1 Laboratory of Epithelial Biology and Disease, Imagine Institute, Université Paris Descartes‐Sorbonne, Paris, France

The paper by Ozisik *et al* investigates associations of Vitamin A and D target genes with renal development and CAKUT pathways. They find that a significant overlap for vitamin A target genes but less so for vitamin D target genes. This fits with previous epidemiological studies on vitamin A and renal size. Overall, I find the paper interesting because it provides insights into non-genetic causes of CAKUT. I have only a few minor points:

- Given that a list of 1086 Vit A target genes but only of 263 Vit D target genes, it is not surprising that less overlap is found for Vit D. This should be commented on.
- It is said in abstract and introduction that only 5% to 20% of CAKUT cases have a monogenic origin. However, it has to be noted that these numbers are only based on whole exome sequencing studies. It is to be expected that whole genome sequencing will discover new monogenic causes.
- The discussion could include more perspectives on how new -omic techniques could be used in the future.

Is the work clearly and accurately presented and does it cite the current literature?

Yes

If applicable, is the statistical analysis and its interpretation appropriate?

I cannot comment. A qualified statistician is required.

Are all the source data underlying the results available to ensure full reproducibility?

Yes

Is the study design appropriate and is the work technically sound?

Yes

Are the conclusions drawn adequately supported by the results?

Yes

Are sufficient details of methods and analysis provided to allow replication by others?

Yes

Reviewer Expertise:

Human genetics, developmental biology, Wnt

I confirm that I have read this submission and believe that I have an appropriate level of expertise to confirm that it is of an acceptable scientific standard, however I have significant reservations, as outlined above.

# Response

**Published:** 2022-04-15

**Authors:** Ozan Ozisik

**Affiliations:** Aix Marseille University, France

We thank the reviewer for the valuable comments. We present the reviewer comments, our answers to them, and quoted texts from the revised manuscript below.

**Given that a list of 1086 Vit A target genes but only of 263 Vit D target genes, it is not surprising that less overlap is found for Vit D. This should be commented on.**

 Considering the overlaps between gene lists, as stated, with less target genes, less genes are expected to overlap with the CAKUT-related gene sets. However, in the manuscript, we do not focus on the exact number of overlapping genes. Indeed, in order to take into account the variable sizes of the different datasets, we used the hypergeometric test. Our focus is hence on the significance of the overlaps considering the dataset sizes, i.e. the observed overlaps as compared to the expected overlaps given the dataset sizes. This means that a small number of overlapping genes could lead to significance for vitamin D overlap with CAKUT-relative genes. However, we did not observe many significant overlaps. The overlaps between vitamin A targets and CAKUT-related gene sets were greater than expectations, which is the reason they are statistically significant. In the revised manuscript we added the explanations for the statistical test.

**It is said in abstract and introduction that only 5% to 20% of CAKUT cases have a monogenic origin. However, it has to be noted that these numbers are only based on whole exome sequencing studies. It is to be expected that whole genome sequencing will discover new monogenic causes.**

 We used a list of causal genes curated from multiple studies. We are not sure if they are all based on whole exome sequencing. However, we agree that whole genome sequencing will help discovering new monogenic causes. We hence added a statement related to the whole genome sequencing to the conclusion:

*"The increased availability of multi-omics technologies, such as whole genome sequencing and metabolomics will certainly open new directions, e.g. by revealing new causal genes or identifying metabolic disturbances due to environmental factors."*

**The discussion could include more perspectives on how new -omic techniques could be used in the future.**

 We agree that we do expect a lot of developments in this area, including whole genome sequencing but also single-cell or spatial transcriptomics, on human, animal models or organoids. In order to mention all these potential technologies while keeping the text focused, we added the following sentence to the conclusion:

*"The increased availability of multi-omics technologies, such as whole genome sequencing and metabolomics will certainly open new directions, e.g. by revealing new causal genes or identifying metabolic disturbances due to environmental factors."*

# Peer review: Reviewer response for version 1

**DOI:** 10.5256/f1000research.54123.r85565

**Published:** 2021-06-25

**Authors:** Elena Menegola

**Affiliations:** 1 Department of Environmental Sciences and Policies, Università degli Studi di Milano, Milan, Italy

The manuscript "Overlap if vitamin A and vitamin D target genes with CAKUT-related processes" is aimed to indirectly evaluate the possible relationship between Vitamin A and D intake imbalance and congenital anomalies of the kidney and urinary tract (CAKUT). The work considers genes reported as dysregulated in CAKUT and key genes involved in different urinary morphogenetic pathways. Both were overlapped with target genes of Vitamin A and Vitamin D (extracted from CTD and from literature: Balmer and Blomhoff, 2002 for Vitamin A, Ramagopalan *et al*., 2010 for Vitamin D). As expected considering the biological activity of the Vitamin A morphogenetic derivative (retinoid acid), significant overlaps are reported in the manuscript between Vitamin A target genes and genes involved in several urinary morphogenetic processes and signalling. Interestingly, significant overlaps were observed between Vitamin A target genes and CAKUT-related genes. As far as Vitamin D is concerned, overlappings were limited to some kidney morphogenetic genes but no overlapping was evidenced with CAKUT-related genes.

 In general, this work can be considered of interest in order to postulate a possible relationship between Vitamin imbalance (mainly Vitamin A) and CAKUT and to suggest that a nutritional evaluation in pregnancy is essential to ensure a physiological development and maternal/fetal health.

 Some suggestions in order to improve the impact of the manuscript:

**Abstract:**

 The abstract should be rewritten in order to better clarify aim and results of the work. In particular, Vitamin D results are lacking.

**Introduction:**

 The aim should be better explained. A brief description for the reason of the choice of literature (Balmer and Blomhoff, 2002 for Vitamin A, Ramagopalan *et al., *2010 for Vitamin D) could enhance the comprehension.

**Methods:**

 Indicate retinoic acid as "the active metabolite of vitamin A" instead of "vitamin A acid". Better explain the statistical analysis.

**Results:**

 Tables are not fully self explicative. I suggest:

- a separate column for p-value;
- write in bold the genes both overlapping with CAKUT and urinary pathways/signalling.

Moreover, (1) in table 2 is not explained.

 Looking in detail at Vitamin A table, four CAKUT-causal genes (BMP4, HNF1B, NRIP1 and RET, overlapping both with CTD and Balmer and Blomhoff) appear as a leitmotiv in different vitamin-A related morphogenetic pathways. In my personal opinion, this result should be stressed and a brief description of functions of BMP4, HNF1B, NRIP1 and RET in kidney and urinary tract development introduced in Results and/or Conclusions.

**Conclusions:**

 Can be rewritten in order to better focus the relevance of the nutritional management during pregnancy in order to ensure both maternal and fetal health. A general consideration should be introduced, considering also the pleiotropic functions of the involved genes.

**References:**

 Please check references, i.e. reference 54 seems not pertinent.

Is the work clearly and accurately presented and does it cite the current literature?

Partly

If applicable, is the statistical analysis and its interpretation appropriate?

I cannot comment. A qualified statistician is required.

Are all the source data underlying the results available to ensure full reproducibility?

Yes

Is the study design appropriate and is the work technically sound?

Yes

Are the conclusions drawn adequately supported by the results?

Partly

Are sufficient details of methods and analysis provided to allow replication by others?

Yes

Reviewer Expertise:

Developmental toxicology

I confirm that I have read this submission and believe that I have an appropriate level of expertise to confirm that it is of an acceptable scientific standard, however I have significant reservations, as outlined above.

# Response

**Published:** 2022-04-15

**Authors:** Ozan Ozisik

**Affiliations:** Aix Marseille University, France

We thank the reviewer for the detailed summary and valuable comments. We present the reviewer comments, our answers to them, and quoted texts from the revised manuscript below.

**Abstract**

**The abstract should be rewritten in order to better clarify aim and results of the work. In particular, Vitamin D results are lacking.**

 We revised the abstract; we clarified our aim and added the results for vitamin D.

*“...In this study, we aimed to investigate whether vitamin A or vitamin D could have a role in CAKUT aetiology. For this purpose we collected vitamin A and vitamin D target genes and computed their overlap with CAKUT-related gene sets. We observed limited overlap between vitamin D targets and CAKUT-related gene sets. We however observed that vitamin A target genes significantly overlap with multiple CAKUT-related gene sets, including CAKUT causal and differentially expressed genes, and genes involved in renal system development. Overall, these results indicate that an excess or deficiency of vitamin A might be relevant to a broad range of urogenital abnormalities.”*

**Introduction**

**The aim should be better explained. A brief description for the reason of the choice of literature (Balmer and Blomhoff, 2002 for Vitamin A, Ramagopalan *et al., *2010 for Vitamin D) could enhance the comprehension.**

 We clarified the aim of the study in the introduction.

*“In this study, we examined whether vitamin A and vitamin D target genes are related to gene sets involved in CAKUT and renal system development. Our aim is to generate hypotheses for the involvement of these vitamins in the disease aetiology.”*

 We used additional datasets from two publications to have independent sources for vitamin target information. Only a small subset of the articles (27 of 1192) reviewed by Balmer and Blomhoff (2002) are reviewed in CTD, and Ramagopalan *et al.* (2010) is not reviewed in CTD. We clarified this in the methods section.

*“As an independent source, we used the data from the study of Balmer and Blomhoff. ^30^ In this study, the authors reviewed published data from 1191 articles to identify genes regulated by retinoic acid (the active metabolite of vitamin A) in humans and other species. Only a small subset of these articles are part of the CTD curation.”*

*“Ramagopalan et al. ^31^ identified 230 genes with significant expression changes in response to vitamin D on lymphoblastoid cell lines using microarrays. This publication is not part of the CTD curation, hence it brings additional and independent information.”*

**Methods**

**Indicate retinoic acid as "the active metabolite of vitamin A" instead of "vitamin A acid". Better explain the statistical analysis.**

 We changed "vitamin A acid" to "the active metabolite of vitamin A".

*“In this study, the authors reviewed published data from 1191 articles to identify genes regulated by retinoic acid (the active metabolite of vitamin A) in humans and other species.”*

 We added the description of the statistical analysis.

*“We computed the overlaps between vitamin target gene sets and the CAKUT gene sets of interest defined previously. After obtaining the overlap results, we calculated the significance of the overlaps using the hypergeometric test . Hypergeometric test calculates the statistical significance of getting k successes in n draws without replacement from a finite population of size N that contains a total of K successes. In our context, k is the number of genes overlapping with a specific gene set, K is the size of that gene set, n is the number of vitamin targets, and N is the background gene set (also known as the reference set). We set N to the number of annotated genes in the database from which we obtained the tested gene set. For the CAKUT causal genes set and the differentially expressed genes set, we used the largest N among the different databases, which corresponds to Gene Ontology. The null hypothesis is that the vitamin target genes are not associated with the gene set and the selection of k genes from that gene set is just a result of simple random sampling.*

*We performed a hypergeometric test for all the gene sets. A consequence of multiple testing is false discoveries in which null hypothesis is rejected erroneously. To control for the false discovery rate, we used the Benjamini-Hochberg correction method (BH adjusted).”*

**Results**

**Tables are not fully self explicative. I suggest:**

- **a separate column for p-value;**

Adding a new column to the table for p-values causes difficulty in layout. We hence added “p-value =” before each value in the parentheses to clarify the table. 

- **write in bold the genes both overlapping with CAKUT and urinary pathways/signalling.**

The overlap of CAKUT causal genes with other gene sets can be interesting for the reader. However, as the aim of the tables in the manuscript is to present overlap of vitamin target genes and CAKUT related gene sets, presenting this extra information might be confusing for the reader. Additionally, the information of overlap between CAKUT causal genes and other pathways will be incomplete and could be misleading as there might be other genes which are common to these but are not vitamin targets. For these reasons, we now provide the overlap of CAKUT causal genes with all the other pathways (without considering whether genes are vitamin targets or not) in a supplementary dataset. This analysis is described in “Supp/SupplementaryAnalyses.odt”, the code for the calculation of this overlap is available as part of overlapAnalysis.py, and the overlap table is presented in “Supp/CAKUTCausalGenesOverlapWithOthers/CAKUTCausalGenesOverlap.csv” in Underlying data and Extended data.

**Moreover, (1) in table 2 is not explained.**

 “(1)”s in table 2 were p-values of 1. We hope that with the addition of “p-value =”, these p-values are clearer now.

**Looking in detail at Vitamin A table, four CAKUT-causal genes (BMP4, HNF1B, NRIP1 and RET, overlapping both with CTD and Balmer and Blomhoff) appear as a leitmotiv in different vitamin-A related morphogenetic pathways. In my personal opinion, this result should be stressed and a brief description of functions of BMP4, HNF1B, NRIP1 and RET in kidney and urinary tract development introduced in Results and/or Conclusions.**

 We agree that these are indeed interesting genes to highlight. We stressed that BMP4, HNF1B, NRIP1 and RET, which are CAKUT causal genes, are vitamin A targets according to both CTD and Balmer and Blomhoff. We added a description of their roles.

*“Four genes (BMP4, HNF1B, RET, NRIP1) appear as particularly interesting as they are CAKUT causal genes and vitamin A targets according to both CTD and Balmer and Blomhoff. BMP4 is a growth factor of the TGF-beta family involved in a wide variety of developmental processes. ^48^ BMP4 inhibits ectopic budding of the ureteric tips and promotes elongation of the ureter. ^49^ RET is a tyrosine-protein kinase receptor, involved in a large variety of cellular processes. RET signaling is important, in particular, for the terminal growth of the nephric duct, and for initial formation and outgrowth of the ureteric bud. ^50^ The Hepatocyte Nuclear Factor 1-beta (HNF1B) is a transcription factor involved in ureteric bud branching and induction of nephrogenesis. It is required for normal S-shaped bodies patterning and subsequent morphogenesis of all nephron segments. ^51^ Finally, Nuclear receptor interacting protein 1 (NRIP1) modulates the activity of nuclear receptors. It has been shown to play roles in renal malformations via deregulations of retinoic acid signaling. ^52^”*

**Conclusions:**

**Can be rewritten in order to better focus the relevance of the nutritional management during pregnancy in order to ensure both maternal and fetal health. A general consideration should be introduced, considering also the pleiotropic functions of the involved genes.**

 We added that our findings indicated the importance of nutritional management during pregnancy.

*“Overall, the significant overlaps observed between vitamin A and CAKUT gene sets indicate that vitamin A might be relevant to a broad range of urogenital abnormalities, pointing out the importance of nutritional management during pregnancy for fetal development.”*

 We thank the reviewer for the suggestion for considering the pleiotropic functions of the involved genes, this can be interesting, however we think that getting into this subject may cause loss of the focus in the conclusion, in which we only state the involvement of vitamin targets in CAKUT related gene sets with the note that our study aims at hypothesis generation.

**References:**

**Please check references, i.e. reference 54 seems not pertinent.**

 We checked the references and we removed the sentence containing this reference.
